# Penis “En Bloc”: From Reproducible Harvesting to Transplantation in a Cadaver study

**DOI:** 10.1016/j.euros.2025.12.010

**Published:** 2025-12-26

**Authors:** Mathieu Fourel, Nicolas Morel-Journel, Lionel Badet, Alain Ruffion, Damien Carnicelli, Philippe Chaffanjon, Gaelle Fiard, Samuel Airoldi, Fabien Boucher, Paul Neuville

**Affiliations:** a Department of Urology, Lille Academic Hospital, University of Lille, Lille, France; b Department of Urology, Hospital Lyon-Sud, Hospices Civils de Lyon, Pierre-Bénite, France; c Department of Urology Surgery and Transplantation, Edouard Herriot Hospital, Lyon, France; d Department of Endocrine and Thoracic Surgery, CHU Grenoble Alpes, University Grenoble Alpes, Grenoble, France; e Laboratory of Anatomy of the French Alps, University Grenoble Alpes, Grenoble, France; f Urology Department, University Hospital Grenoble, Grenoble, France; g Plastic and Reconstructive Surgery Department, Croix Rousse Hospital, Hospices Civils de Lyon, Lyon, France

**Keywords:** Vascularized composite allograft, Penile transplant, Cadaver, Harvesting, Transplantation

## Abstract

**Background and objective:**

Our aim was to assess the reproducibility of en bloc penile harvesting with a focus on the vascular structures to determine whether the procedure could be performed while preserving critical vascular supply.

**Methods:**

A single-center, prospective cadaver study was conducted from November 2023 to October 2024 using 15 male cadavers, a number determined a priori. The main outcome criterion was successful harvesting and transplantation. This was defined as a harvest that included the entire corpora cavernosa, the urethra up to the subprostatic region, the pudendal nerves, the external pudendal arteries to their origin, the external pudendal veins to their termination, and the internal pudendal arteries to their origin, the deep dorsal vein. Transplantation was considered successful if arterial, venous, urethral, and nerve anastomoses were possible.

**Key findings and limitations:**

Thirteen harvests were deemed successful and were associated with 13 transplantations. The external pudendal vessels were anastomosed to the superficial femoral artery, the great saphenous vein, or one of its accessory branches. The internal pudendal artery was anastomosed to either the external iliac artery or the deep inferior epigastric artery. The urethra, pudendal nerves, and deep dorsal vein were anastomosed with their respective counterparts in the recipient. The main study limitation is the cadaver setting.

**Conclusions and clinical implications:**

Our study confirms that harvesting of the entire penile structure—including the external pudendal vessels, deep dorsal vein, pudendal nerves, internal pudendal arteries, and urethra—is both feasible and reproducible in a cadaver model. Furthermore, use of such a graft appears to be anatomically achievable.

**Patient summary:**

In a cadaver study, we demonstrated that our technique for harvesting the entire penis is feasible and reproducible. This could expand the range of conditions for which a penis transplant is possible.

## Take Home Message

An “en bloc” penile harvest, including the internal pudendal artery to its origin, is technically feasible. A graft using such a transplant appears to be feasible and would allow for preservation of the recipient vessels commonly used in phalloplasty procedures.

## 1 Introduction

The period from 1990 to 2000 saw significant advances in composite tissue allotransplantation [1], particularly in the area of male genitalia transplantation. In 2006, Hu et al. [2] performed the first penile allotransplantation, although the graft survived for only 14 d. This pioneering procedure was followed by four additional penile transplantations (PTs) worldwide that yielded encouraging functional outcomes, including recovery of normal urinary function, satisfactory erectile function, and restoration of both tactile and erogenous sensation [3]. However, it is important to emphasize that grafts obtained from segmental penile harvests are not uniform, and successful PT requires a well-preserved and anatomically suitable recipient stump. As with all transplant procedures, PT carries inherent risks and may result in graft loss [4]. In such cases, reconstruction via phalloplasty remains a viable option. However, if the initial graft utilized vascular structures commonly used in free flap phalloplasties [3], options for subsequent reconstruction may be significantly limited.

To broaden surgical indications and reduce the number of anastomoses required, the concept of harvesting the entire penile structure has emerged. This approach includes all major penile components—the corpora cavernosa, urethra, penile shaft, and dorsal nerves—and involves harvesting the primary vascular supply at its origin, specifically the internal and external pudendal arteries. Proof of concept has successfully been established in cadaver models [5], [6], [7], [8]. However, limited sample availability [7] and inconsistent reporting of vascular anatomy have hindered reproducibility.

The objective of this study was to assess the reproducibility of en bloc penile harvesting, with a particular focus on the vascular structures, to determine whether the procedure could be performed in a cisgender patient while preserving critical vascular supply.

## 2 Patients and methods

### 2.1 Design and ethics

This was a single-center, prospective, cadaver study conducted from November 2023 to October 2024 at the Anatomy Laboratory of the French Alps, part of the University of Grenoble Alpes. The study was carried out following approval from the ethics committee of the Grenoble Alpes body donation center and was supported by the French Urology Association.

### 2.2 Population

This study involved 15 male cadavers preserved via formaldehyde injection (solution concentration between 1% and 2%). The two-stage Simon method [9] was used for study design and to perform an a priori sample size calculation. A successful harvesting rate >70% (p0) was considered sufficient to deem the technique reproducible. To achieve statistical power of 90% and a type I error rate of 10%, a total of 15 cadavers was required. The first stage included nine cadavers. If eight or nine harvests were successful, the study would proceed with the inclusion of six additional cadavers. Otherwise, the study would be halted, and the causes of failure would be analyzed and documented. If the study continued, the final evaluation of harvesting success and reproducibility would be based on all 15 cadavers. Success for 13 or more harvests was the criterion for reproducibility of the technique.

There were no specific inclusion or exclusion criteria; inclusion was based on the availability of bodies in the laboratory. The subjects may have undergone previous dissections for other purposes, but the genital and pelvic regions were always preserved.

### 2.3 Outcomes

The primary outcome criterion was successful harvesting and transplantation. A harvest was considered successful if it included: the entire corpora cavernosa, the urethra up to the sub-prostatic region, the pudendal nerves, the external pudendal arteries (EPAs) to their origin, the external pudendal veins (EPVs) to their termination, the internal pudendal arteries (IPAs) to their origin, and the deep dorsal vein (DDV). Transplantation was considered successful if arterial, venous, urethral, and nerve anastomoses were feasible.

Secondary outcome criteria included the duration of harvesting (measured in minutes), measurement of vessel length (in centimeters), and measurement of vessel caliber (in millimeters).

### 2.4 Statistical analysis

Results are reported as the median and interquartile range (IQR) for quantitative variables and as the frequency and percentage for qualitative data.

### 2.5 Dissection protocol

The technique for en bloc penile harvesting has previously been described [8]. It can be summarized as the following steps: harvesting of the external pudendal vessels along with a patch of the femoral artery and the great saphenous vein; performing an iliac laparotomy; performing a pubic osteotomy; harvesting of the IPA up to its internal iliac origin; and explantation of the graft.

At least two surgeons participated in each procedure, with one surgeon (M.F.) present for all dissections.

## 3 Results

### 3.1 Harvesting of the graft

**Fig. 1:**
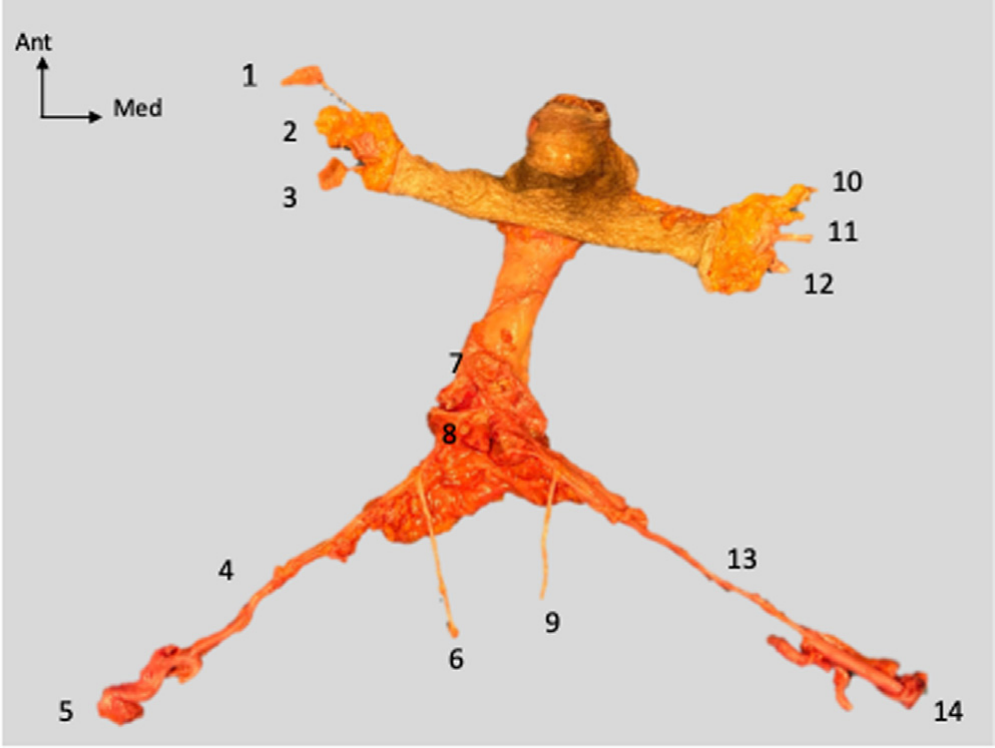
“En bloc” harvesting of the penis. (1) Left inferior external pudendal artery with patch. (2) Left external pudendal vein. (3) Left superior external pudendal artery with patch. (4) Left internal pudendal artery. (5) Left common iliac artery. (6) Left pudendal nerve. (7) Deep dorsal vein of the penis. (8) Urethra. (9) Right pudendal nerve. (10) Right inferior external pudendal artery with patch. (11) Right external pudendal vein. (12) Right superior external pudendal artery with patch. (13) Right internal pudendal artery. (14) Right common iliac artery. Ant = anterior; Med = medial.

The 15 dissections were successfully completed according to the pre-established protocol. Of these, 13 harvests were classified as successful. In these 13 cases, all of the following structures were present: both IPAs, including their origin from the internal iliac arteries; both pudendal nerves; the DDV of the penis; the urethra; the two corpora cavernosa; the EPA(s) with the femoral artery patch; and the EPV(s) with the great saphenous vein (Fig. 1).

The characteristics available for the subjects are presented in Table 1. The median age was 80 yr (IQR 75–87.5) and median body mass index was 26 kg/m^2^ (IQR 23.9–27.7). The median time for a successful harvest was 199 min (IQR 190–210.5).

**Table 1:** Characteristics of the penile harvests (*n* = 15)

Parameter	Median (IQR)
Age (yr)	80 (75–87.5)
Body mass index (kg/m^2^)	26 (23.9–27.7)
Time since death (d)	20 (16–28)
Harvest duration (min)^a^	199 (190–210.5)

IQR - interquartile range.

a *n* = 13.

The measurements obtained during the harvests were summarized in Table 2, Table 3. The EPA most commonly originated from the superficial femoral artery and was single in 86.7% of cases (13/15) on the left side, and in 73.3% of cases (11/15) on the right side. The EPV, which drained into the great saphenous vein, was single in 66.7% of cases (10/15) on the left, and in 53.3% of cases (8/15) on the right. The median diameter of the EPA, when singular, was 2.6 mm (IQR 1.8–2.9) on the left and 2.9 mm (IQR 2.1–3.3) on the right. The median diameter of the EPV was 3.4 mm (IQR 2.7–3.9) on the left and 2.7 mm (IQR 2.4–3.9) on the right. The median length of these vessels when singular and measured from their origin to the root of the penis was 12 cm (IQR 10.5–13.1) on the left and 11.9 cm (IQR 11.3–13) on the right for the EPA, and 9.2 cm (IQR 8.9–10.2) on the left and 10.6 cm (IQR 9.1–11.7) on the right for the EPV.

**Table 2:** Characteristics of the external pudendal vessels on either side of harvest

Parameter	Left side (*n* = 15)	Right side (*n* = 15)
**Arteries**		
Number of arteries, *n* (%)		
1 artery	13 (86.7)	11 (73.3)
2 arteries	2 (13.3)	4 (26.7)
Origin, *n* (%)		
Common femoral artery	5 (33.3)	4 (26.7)
Superficial femoral artery	10 (66.7)	11 (73.3)
Median caliber, mm (IQR)		
Single trunk	2.6 (1.8–2.9)	2.9 (2.1–3.3)
Double trunk		
Superior trunk	1.9 (1.6–2.1)	1.7 (1.4–2.3)
Inferior trunk	1.9 (1.8–1.9)	1.9 (1.7–2.0)
Median length, cm (IQR)		
Single trunk	12 (10.5–13.1)	11.9 (11.3–13)
Double trunk		
Superior trunk	12.4 (12.3–12.4)	12.8 (12.1–12.3)
Inferior trunk	13.1 (12.9–13.2)	12.9 (12.4–13.6)
**Veins**		
Number of veins, *n* (%)		
1 vein	10 (66.7)	8 (53.3)
2 veins	5 (33.3)	7 (46.7)
Termination, *n* (%)		
Great saphenous vein	15 (100)	15 (100)
Median caliber, mm (IQR)		
Single trunk	3.4 (2.7–3.9)	2.7 (2.4–3.9)
Double trunk		
Superior trunk	1.9 (1.8–3.3)	2.8 (2.8–3)
Inferior trunk	2 (1.7–2.2)	2.3 (2.0–2.8)
Median length, cm (IQR)		
Single trunk	9.2 (8.9–10.2)	10.55 (9.1–11.7)
Double trunk		
Superior trunk	11.7 (9.4–12)	9.7 (8.1–10.8)
Inferior trunk	11.3 (9.9–11.9)	8.5 (7.9–10.3)

IQR = interquartile range.

**Table 3:** Characteristics of the internal pudendal arteries by side of harvest

	Left side (*n* = 13)	Right side (*n* = 14)
Median caliber, mm (interquartile range)		
Origin internal iliac artery	8.9 (8.4–10.4)	9.1 (8.3–10.4)
Origin internal pudendal artery	2.9 (2.3–3.0)	2.8 (2.1–3.1)
Median length, cm (interquartile range)		
Origin internal iliac artery	21.5 (21–22)	22.3 (21.5–22.8)
Origin internal pudendal artery	13 (11.4–14.9)	15.4 (13.6–17.4)

Two IPA segments were measured: (1) from the iliac bifurcation (representing the maximum possible length) and (2) from the origin of the IPA to the cavernous bodies. The analysis also included data on failures when available. On the left, the median maximum length was 21.5 cm (IQR 21–22) and the median diameter was 8.9 mm (IQR 8.4–10.4). On the right, the median maximum length was 22.3 cm (IQR 21.5–22.8) and the median diameter was 9.1 mm (IQR 8.3–10.4).

### 3.2 Graft

Out of the 15 harvests, 13 grafts were successfully performed, with two excluded because of prior harvesting failure.

Recipient preparation involved severing of the corpora cavernosa at the level of the pubic symphysis to create an appropriate stump. The dorsal vessels and nerves were then isolated and dissected free from the tunica albuginea of the corpora cavernosa, as were the urethra and the corpus spongiosum. The corpora cavernosa, urethra, and corpus spongiosum were incised dorsally to allow for spatulation.

**Fig. 2:**
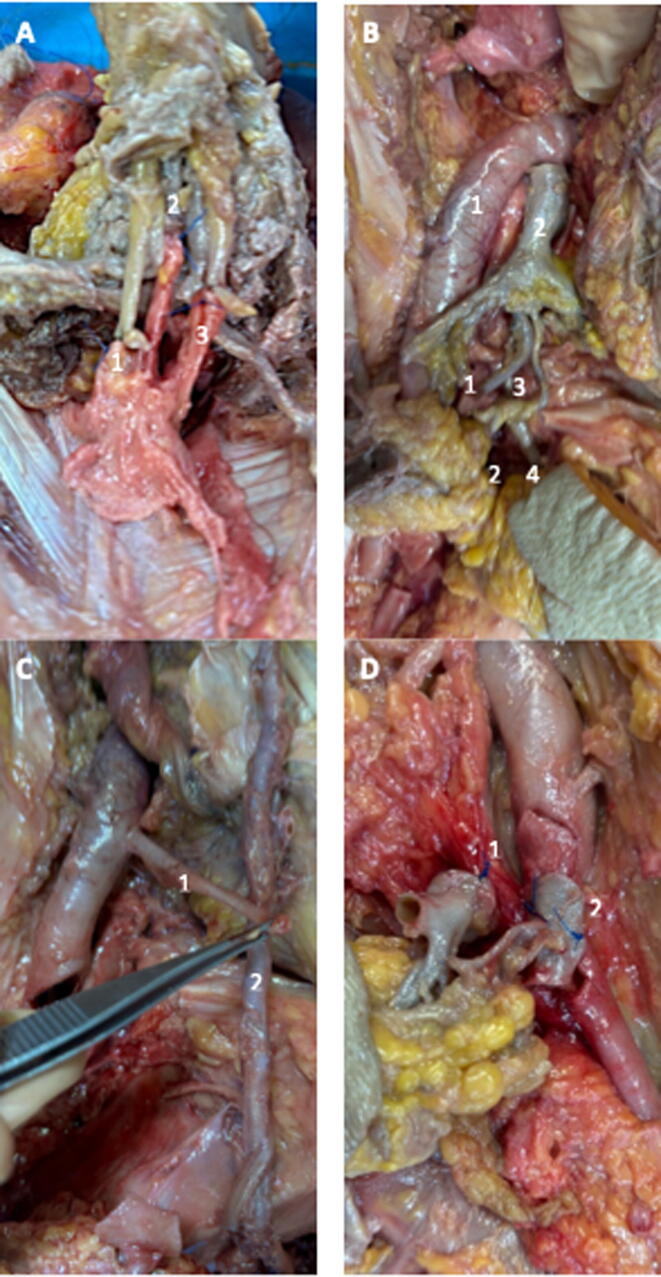
Photographs illustrating key stages of the graft procedure. (A) Anastomosis of the deep dorsal vein and dorsal nerves. (1) Anastomosis of the donor’s left dorsal nerve with that of the recipient. (2) Anastomosis of the donor’s deep dorsal vein with that of the recipient. (3) Anastomosis of the donor’s right dorsal nerve with that of the recipients. (B) Potential anastomosis between the donor’s internal iliac artery and the recipient’s external iliac artery. (1) Right external iliac artery of the recipient. (2) Donor’s internal iliac artery, continuing into the internal pudendal artery. (C) Potential anastomosis of the donor’s internal pudendal artery with the recipient's inferior epigastric artery. (1) Inferior epigastric artery. (2) Internal pudendal artery. (D) Anastomosis of the left external pudendal vessels. (1) Anastomosis of the donor’s great saphenous vein with that of the recipient. (2) Anastomosis of the donor’s external pudendal artery with that of the recipient.
• The donor’s pudendal nerves were anastomosed to the recipient’s dorsal penile nerves (Fig. 2A).
• For the donor’s IPA, two reconstruction strategies were possible owing to their generous length:
  o An anastomosis between the donor’s internal iliac artery patch and the recipient’s external iliac artery was feasible via an iliac laparotomy approach (Fig. 2B).
  o Alternatively, an anastomosis between the donor’s IPA and the recipient’s deep epigastric artery could be performed using the same approach (Fig. 2C).
• The EPV(s) were anastomosed to the recipient’s great saphenous vein via the donor’s great saphenous vein (Fig. 2D).
• The EPA(s) were anastomosed to the femoral artery (Fig. 2D).

The following anastomoses were performed:

• A ventral, proximal cavernotomy of the donor’s corpora cavernosa was performed to allow a terminolateral anastomosis using two running sutures.
• The donor’s urethra and corpus spongiosum were trimmed to an appropriate length and dorsally spatulated for the recipient urethra, and ventrally for the donor urethra, to facilitate a tension-free end-to-end anastomosis.
• The donor’s DDV was anastomosed to the recipient’s DDV (Fig. 2A).

Following these anastomoses, the penis was secured to the periosteum of the pubic symphysis using several separate stitches placed through the proximal tunica albuginea of the corpora cavernosa. Finally, the skin was closed using staples.

## 4 Discussion

Our study presents an en bloc penile harvesting technique and assesses its reproducibility via iterative cadaveric dissections, demonstrating favorable reproducibility outcomes. In addition, this evaluation was complemented by a graft implantation assessment, supporting the feasibility of this approach for PT while preserving recipient vascular structures—an important consideration for optimizing long-term care and enabling potential future reconstructions. We also provide detailed measurements of the pudendal vessels and describe their anatomical variability.

Preliminary studies suggested the feasibility of en bloc penile harvesting [5], [6], [7], consistent with the initial findings we reported for our innovative approach [8].

Studies evaluating the length and diameter of the IPA are scarce; to the best of our knowledge, only one cadaver study [5] and one radiological study using computed tomography (CT) imaging [10] have conducted such measurements. The average diameter at the origin of the IPA was 4 mm in the cadaver study by Cristofari et al. [5], who did not differentiate between sides. By contrast, the CT-based study by Selvaggi et al. [10] revealed a mean diameter of 2.1 mm on the left and 2.4 mm on the right. While such discrepancies may arise from differences in methodology—namely a cadaver setting versus in vivo CT imaging—it is noteworthy that our median diameter falls between these values, at approximately 3 mm. In the same studies, length measurements varied more widely: median length was 10.5 cm on the left and 11.6 cm on the right in the CT study [10], and 15 cm in the cadaver study [5]. Length assessment is generally more challenging via CT imaging, which may account for the substantial differences in comparison to our measurements, which are approximately double those previously reported. In addition, once the artery is fully dissected and detached from surrounding tissues, a degree of passive elongation, even in cadaver specimens, may contribute to an apparent increase in length.

We systematically identified the external pudendal vessels in our study, and primarily found one to two EPAs and one to two EPVs per side. The number of these vessels varies across studies without exceeding two, consistent with our findings [11], [12], [13], [14], [15], [16]. Regarding vessel caliber, our findings align with the literature, with diameters between 2 mm and 3 mm for a single external pudendal trunk, and slightly smaller calibers when two trunks are present [11], [13], [14], [15]. There is limited information available regarding the length of these vessels. However, Cristofari et al. [5] reported an average arterial length of 2.5 cm and venous length of 2.0 cm, which is significantly shorter than the ∼12 cm observed in our study. This discrepancy may be explained by substantial differences in measurement methodology: we measured from the vessel origin to the base of the penis, whereas Cristofari et al. did not specify their measurement method.

One important potential advantage of en bloc penile harvesting is the preservation of vessels that could be used for subsequent phalloplasty or PT in the event of graft failure. In our view, the selection of recipient vessels for graft implantation should prioritize vascular preservation to ensure that subsequent reconstruction via phalloplasty remains feasible if needed, which is a consideration not previously emphasized in PT reports [3]. The detailed selection of recipient vessels is rarely discussed in the phalloplasty literature; commonly described options include the deep inferior epigastric vessels and the great saphenous vein [17], the EPA [14], the femoral artery [18], [19], [20], [21], and the medial or lateral circumflex femoral arteries [22], [23]. In our phalloplasty experience, we predominantly use the deep inferior epigastric vessels, which therefore should be preserved. Our proposed PT approach—using the femoral vessels and/or the great saphenous vein (or one of its accessory branches) for anastomosis with the external pudendal vessels, and the external iliac arteries or deep inferior epigastric arteries for anastomosis with the IPA—ensures effective vascular preservation by sparing vessels commonly required for phalloplasty.

Venous drainage from the erectile bodies is primarily provided by the DDV [24], which must be anastomosed during transplantation. If the recipient’s DDV is unavailable or unusable, an alternative anastomosis between the donor’s DDV and the recipient’s inferior epigastric vein is feasible [25], [26]. Use of the great saphenous vein is also a viable option, and is common in phalloplasty procedures [17].

Regarding arterial anastomosis, use of both IPAs could theoretically allow a single anastomosis via a Y-shaped reconstruction. While this approach could simplify the procedure, it would probably increase the risk of severe vascular complications in the event of arterial thrombosis, and is therefore not favored in our view.

Performing ventral cavernotomy for anastomosis of the donor’s corpora cavernosa is justified by the need to preserve the penile neurovascular pedicle, which runs dorsally. An anastomosis between the donor’s and recipient’s corpora cavernosa should be systematically attempted, even in the context of gender-affirming surgery, in which the clitoral corpora cavernosa may be used. This approach is supported by favorable outcomes reported for cases of penile reimplantation following traumatic amputation, particularly for macrosurgical reimplantation, for which corpora cavernosa anastomosis alone—without arterial reconstruction—has been associated with the return of erectile function [27], [28], [29].

Our study has several limitations, some of which are inherent to the cadaver setting, which limits the applicability of our findings to living, heart-beating bodies. In addition, only en bloc penile harvesting was performed, and the potential interaction of this procedure with other procurement needs—such as harvesting of the iliac vessels or ureters in the context of multiorgan donation—remains to be evaluated in clinical settings. Donor selection was also challenging because information regarding pelvic history was often incomplete or unavailable. While we provided precise measurements of the length and diameter of the pudendal vessels, the condition of the cadaver may affect vascular dimensions in comparison to in vivo measurements. Furthermore, although reproducibility was demonstrated according to a pre-established protocol, the harvesting technique did not yield consistent success in all cases. In our study, two harvests failed. The first case probably involved a history of pelvic radiation, as suggested by skin markings and fibrotic fusion of pelvic tissues. While dissection of the external pudendal vessels was possible, dissection of penile, pelvic, and internal pudendal tissues could not be completed. The second failure was of a technical nature, involving inadvertent transection of the left IPA at the base of the penis. These harvesting procedures were performed on relatively elderly patients, many of whom exhibited calcification of the external and internal pudendal arteries. Dissection of the IPA may have been facilitated by the ease of palpation, a factor that might not be present during cold-phase dissection in actual clinical procurement. Nevertheless, the confirmation of feasibility and demonstration of reproducibility of the en bloc penile harvesting procedure provide confidence in its potential applicability in clinical settings.

## 5 Conclusions

Our study confirms that harvesting of the entire penile structure—including the external pudendal vessels, DDV, pudendal nerves, IPA, and urethra—is both feasible and reproducible in a cadaver model. Furthermore, use of such a graft appears to be anatomically achievable. On the basis of these findings, this technique may broaden the possibilities for PT while preserving the key recipient vessels commonly used in free flap phalloplasty. The next phase of our research program will evaluate this technique in artificially perfused cadavers to better replicate clinical conditions.

  ***Author contributions***: Mathieu Fourel had full access to all the data in the study and takes responsibility for the integrity of the data and the accuracy of the data analysis.

  *Study concept and design*: Fourel, Neuville, Morel-Journel, Boucher.

*Acquisition of data*: Fourel, Neuville, Morel-Journel.

*Analysis and interpretation of data*: Fourel, Neuville, Morel-Journel.

*Drafting of the manuscript*: Fourel, Neuville.

*Critical revision of the manuscript for important intellectual content*: Neuville, Morel-Journel, Carnicelli.

*Statistical analysis*: Fourel.

*Obtaining funding*: Fourel, Neuville, Morel-Journel, Ruffion.

*Administrative, technical, or material support*: Badet, Ruffion, Chaffanjon, Fiard, Airoldi, Boucher.

*Supervision*: Neuville, Morel-Journel, Carnicelli.

*Other*: None.

  ***Financial disclosures:*** Mathieu Fourel certifies that all conflicts of interest, including specific financial interests and relationships and affiliations relevant to the subject matter or materials discussed in the manuscript (eg, employment/affiliation, grants or funding, consultancies, honoraria, stock ownership or options, expert testimony, royalties, or patents filed, received, or pending), are the following: None.

  ***Funding/Support and role of the sponsor*:** This study was supported by Association Française d’Urologie. The sponsor played a role in the design and conduct of the study.
